# Editorials

**Published:** 1908-09

**Authors:** 


### EDITORIALS

The Business Office of the JOURNAL-RECORD is 1 1-2 to 5 1-2 South Broad St.
The Editorial Office is 1014-15 Century Bldg.
Address all Business Communications to Journal-Record of Medicine, 1 1-2 to 5 1-2 South Broad Street.
Make remittances payable to THE JOURNAL-RECORD OF MEDICINE.
On matters pertaining to the Editorial and Original communications, address Edgar G. Ballenger, M. D., Atlanta, Ga.
Reprints of original articles will be furnished at cost price. Requests for the same should always be made in THE MANUSCRIPT.
We will present, postpaid, on request, to each contributor of an original article, twenty (20) marked copies of THE JOURNAL-RECORD OF MEDICINE containing such article.

### OPENING OF THE MEDICAL COLLEGES.

Next week the College of Physicians and Surgeons, and the Atlanta School of Medicine will begin their fall term, and it is confidently predicted that there will be a large crowd of students in attendance at both of the colleges. The excellent equipment, modern in every respect, the corps of able professors and the abundant hospital facilities enable Atlanta to offer many and distinct advantages to medical students Within the last few years both of these colleges have built large commodious quarters in which they are amply able to give thorough training in all of the medical branches. The high stand recently taken by the graduates of the Atlanta schools in passing the various state boards of medical examiners is unequivocal evidence of the excellence of the course of training. It is with pleasure we note the increase in the preliminary requirements, and we trust the standard may be raised still higher as the common schools of Georgia improve.

### MUSQUITOES.

The $2,000 appropriated by the city for the purpose of fighting musquitoes has been exhausted and as a result Atlanta is now having an epidemic of this disease-spreading pest. As long as the fund lasted and the campaign against the musquitoes was waged, the city remained practically free from them. The health department has announced that its efforts along this line will now have to cease as the funds have given out entirely. Dr. Kenedy, city health officer, states that the fight should be continued six weeks longer, as September and October are two of the most important months for this kind of work. It is earnestly hoped that an appropriation will be made to continue this fight.

---

### DR. BOLAND'S SKETCH OF HENRY GRAY.

The biographical sketch of a great man in medicine is always a source of interest and instruction to the physician, and the September number of the American Journal of the Medical Sciences contains a valuable contribution of this nature, from the pen of Dr. Frank K. Boland, of Atlanta. The article is entitled "Henry Gray, Anatomist: An Appreciation," and shows the result of considerable investigation.

Although Gray's Anatomy is one of the best known of all medical books, information concerning the life of the author heretofore has been very scant. The reasons for this, as Dr. Boland explains, have been Gray's premature death and the fact that he left no direct descendents to record the particulars of his career. Apparently, this sketch exhausts all that can be learned of the brilliant young anatomist, after a lapse of a half century, and the profession is under obligations to Dr. Boland for his services as a biographer.

Henry Gray was born in London in 1827, and died of confluent small pox in 1861, at the age of thirty-four. He began the study of medicine at St. George's Hospital Medical School at the age of eighteen, and his student and professional course to success was uninterrupted. There is no truth in the story that he was a poor student and failed in some of his examinations. The facts, as obtained from competent authorities, tends to prove the very opposite. He was awarded several important prizes as a student, and became a member of the Royal Society at the remarkably early age of twenty-five.

The first edition of his "Anatomy, Descriptive and Surgical" appeared in 1858, when Gray was but thirty-one years of age. Although this work was his masterpiece, he wrote many noteworthy papers on other subjects. The Anatomy was a success from the first, notwithstanding the critics were by no means unanimous in their praise. Some charged the author with borrowing from Quin and Sharpey's work, which hitherto had been the most popular English text-book. There charges were not sustained, and it became well-established that Gray described only the dissections that were actually before his eyes.

The potrait of Gray accompanying the sketch, which was copied from a picture at St. George's Hospital, shows him to have been a strickingly handsome young man, with a high forehead and a powerful chin. There is but little recorded of the personal side of his character, and nothing said of his ability as a diagnostician or operator. The plan of his professional career, which is worthy of emulation, seems to have been first to ground himself thoroughly in the fundamental branches of anatomy and pathology, after which he would be equipped for the best possible work in the field of practical surgery.

Dr. Boland's summary, in these words, states very fairly the estimate in which Gray and his work are held today: "From this brief study of the life of Henry Gray, which was undertaken with the purpose of determining his true place in medical history, I am happy to conclude that he is entitled to the fame that is his. Although confessedly the glamor of renown has been augmented by clever illustrators, able editors, and progressive publishers, yet we could not fairly snatch away a single ray when we consider the part he played in the time allotted him. These factors are always instrumental in the success of such work. Gray's genius, like that of many others, consisted of hard work and singleness of purpose; could he have lived long enough to carry this with his spirit of investigation farther into medical science, it is reasonable to believe that he would have a name as great as surgeon as it is as anatomist and teacher."

It must not be supposed that no good work on anatomy existed before *Gray* made its appearance. The excellent books of English and Franch authors had strong following, and the new work was pitted against keen competition. But Gray felt that there was room for improvement in the old treatises, particularly in the matter of arrangement and illustrations. He had a good conception of the principles of pedagogy, and desired, in so far as he could, to smooth for his students the hard road to anatomical knowledge. It was not the presentation of any new anatomical discoveries, but the clearer and more systematic presentation of old ones, backed strongly by unprecedented drawings, which secured for his book its phenomenal run.

